# Restriction by APOBEC3 proteins of endogenous retroviruses with an extracellular life cycle: *ex vivo *effects and *in vivo *"traces" on the murine IAPE and human HERV-K elements

**DOI:** 10.1186/1742-4690-5-75

**Published:** 2008-08-14

**Authors:** Cécile Esnault, Stéphane Priet, David Ribet, Odile Heidmann, Thierry Heidmann

**Affiliations:** 1 Unité des Rétrovirus Endogènes et Eléments Rétroïdes des Eucaryotes Supérieurs, CNRS UMR 8122, Institut Gustave Roussy, 39 rue Camille Desmoulins, F-94805 Villejuif, and Université Paris-Sud, Orsay, F-91405, France; 2 Architecture et Fonction des Macromolécules Biologiques, CNRS UMR 6098, ESIL case 925, F-13288 Marseille Cedex 9, France; 3 Unité des interactions Bactéries-Cellules, INSERM U604, INRA USC2020, Institut Pasteur, 25 rue du Dr Roux, F-75024 Paris Cedex 15, France

## Abstract

**Background:**

APOBEC3 cytosine deaminases have been demonstrated to restrict infectivity of a series of retroviruses, with different efficiencies depending on the retrovirus. In addition, APOBEC3 proteins can severely restrict the intracellular transposition of a series of retroelements with a strictly intracellular life cycle, including the murine IAP and MusD LTR-retrotransposons.

**Results:**

Here we show that the IAPE element, which is the infectious progenitor of the strictly intracellular IAP elements, and the infectious human endogenous retrovirus HERV-K are restricted by both murine and human APOBEC3 proteins in an *ex vivo *assay for infectivity, with evidence in most cases of strand-specific G-to-A editing of the proviruses, with the expected signatures. *In silico *analysis of the naturally occurring genomic copies of the corresponding endogenous elements performed on the mouse and human genomes discloses "traces" of APOBEC3-editing, with the specific signature of the murine APOBEC3 and human APOBEC3G enzymes, respectively, and to a variable extent depending on the family member.

**Conclusion:**

These results indicate that the IAPE and HERV-K elements, which can only replicate via an extracellular infection cycle, have been restricted at the time of their entry, amplification and integration into their target host genomes by definite APOBEC3 proteins, most probably acting in evolution to limit the mutagenic effect of these endogenized extracellular parasites.

## Background

The APOBEC family of cytosine deaminases includes numerous members that can deaminate cytosine to uracil within DNA and/or RNA molecules. Among these enzymes, the APOBEC3 sub-family has been discovered when human APOBEC3G (hA3G) was reported to restrict HIV replication ([1]; reviewed in [2]). Human hA3G has been shown to trigger extensive deamination of cytosine in the negative viral DNA strand during reverse transcription and to lead to deleterious G-to-A mutations considered as the hallmark of APOBEC3-editing activity. Subsequently, several other human APOBEC3 proteins – including APOBEC3A (hA3A) [3], APOBEC3B (hA3B) [4,5], APOBEC3C (hA3C) [5], APOBEC3DE (hA3DE) [6], APOBEC3F (hA3F) [7-9] and APOBEC3H (hA3H) [10] – have been shown to exhibit antiviral effects against a variety of viruses, including numerous retroviruses – i.e. HIV, SIV, MLV, HTLV and foamy viruses –, hepatitis B virus and adeno-associated virus (AAV) (for review [11]). In contrast to humans, the mouse genome encodes only one APOBEC3 (mA3) protein, which, like human APOBEC3 proteins, displays antiviral effects [12]. Aside from the antiviral function of APOBEC3 proteins against exogenous viruses, some inhibitory effects have been reported on intracellular targets (for review [2]) and several studies support the notion that the primary function of APOBEC3 proteins could be to prevent the propagation of mobile elements. Indeed, mammalian genomes have accumulated numerous transposable elements which account for > 45% of the genomic DNA [13,14]. These elements can be grouped into two main classes: the strictly intracellular non-LTR (Long Terminal Repeat) retrotransposons, namely long interspersed nuclear elements (LINEs) and short interspersed nuclear elements (SINEs), which account for ~30% of each mammalian genome, and the LTR-containing retroelements (including the endogenous retroviruses, ERVs), accounting for ~10% of the genomes and closely related to retroviruses. The life cycle of ERVs includes the formation of virus-like particles (VLPs) that, in several instances – but not systematically – can remain strictly intracellular as observed for the well-characterized murine intracisternal A-particle (IAP) and MusD elements (the so-called "intracellularized" ERVs, [15-18]), or that can bud at the cell membrane for an extracellular cycle as observed for the recently identified murine intracisternal A-particle-related envelope-encoding (IAPE; [18]) and the human endogenous retrovirus HERV-K(HML2) elements [19,20]. Although most of these elements are no longer active due to the accumulation of inactivating mutations, some of them are still functional and have been cloned, thus allowing direct *ex vivo *assay of the effect of APOBEC proteins on their mobility. Accordingly, several APOBEC3 proteins, including hA3A, hA3B, hA3C and hA3F have been demonstrated to restrict the retrotransposition of the human LINE-1 (L1) elements [3,21,22], as well as the L1-dependent transposition [23] of the human *Alu *SINE elements [24]. Moreover, although no effect on the retrotransposition of L1 elements was observed in the presence of hA3G [21,25-27], reports have shown that hA3G can prevent the retrotransposition of *Alu *elements [27,28] by sequestering *Alu *RNAs in cytoplasmic high-molecular-mass (HMM) ribonucleoprotein complexes [28]. Similarly, the cloning of active copies for the intracellular murine IAP and MusD elements [15,17] made possible to demonstrate susceptibility of these retroelements to murine APOBEC3 and to most of the human APOBEC3 proteins [24,26,29]. In addition, *in silico *analyses of the naturally present genomic copies of these elements in the murine genome have revealed "traces" of APOBEC3 editing on these elements ([26]; see also [30]), thus supporting the physiological relevance of the observed *ex vivo *assays, and the genomic impact of APOBEC3 protein activity.

Here we take advantage of the recent identification of the infectious progenitor of the intracellularized IAP retrotransposon, namely IAPE, to analyze the possible restriction of a *bona fide *murine ERV, in a state close to that at the time of its initial endogenization step when the element still behaved as an infectious retrovirus, having not yet reached its highly adapted "intracellularized" state [18]. In parallel, we performed a similar analysis on the human progenitor of the HERV-K(HML2) family members that we had "reconstituted", resulting in the *Phoenix *element which proved to be a *bona fide *endogenous retrovirus, the element being able to enter cells by infection and integrate with all the characteristic features of the genomic copies presently found in the human genome [19]. These two functional human and murine "extracellular" ERVs were used to assess the effects of APOBEC3 proteins on mammalian endogenous retroviruses in appropriate *ex vivo *assays, and refined *in silico *analyses of the naturally present copies of these elements in their target host genomes finally unambiguously demonstrated "traces" of APOBEC3 editing, with identifiable signatures. Altogether, the data show that APOBEC3 proteins play a role not only on the intracellular retrotransposons found in humans and mice, but also on their retroviral "progenitors" endowed with an extracellular life style, thus *de facto *filling the gap between the described effects of APOBEC3 proteins on *bona fide *exogenous retroviruses on the one hand and intracellular retroelements on the other.

## Results and discussion

### Restriction of murine and human infectious ERVs by APOBEC3 proteins

**Figure 1:**
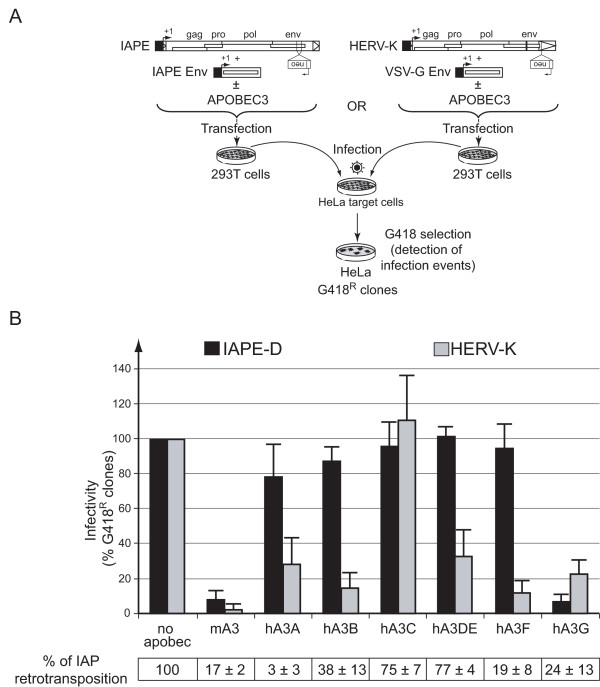
Murine and human APOBEC3 proteins inhibit endogenous retroviruses. (A) Rationale of the assay for detection of infection events by endogenous retroviruses in the presence of APOBEC3 proteins. The IAPE-D and HERV-K elements used in the assay are marked with the *neo *reporter gene – inserted in reverse orientation – and carry their own functional genes, except for the *env *gene which is supplied in *trans*, thus allowing only for single rounds of infection. Human 293T cells are co-transfected with the indicated expression vectors for APOBEC3 family members, the supernatants collected 2-days post-transfection to infect HeLa target cells, and infection events detected upon G418 selection. (B) Analysis of the activity of murine and human APOBEC3 proteins on the indicated endogenous retroviruses. Viral titers are given as percentages relative to a control (no apobec: expression vector with a nonfunctional hA3G; 622 and 549 G418^R ^clones/ml for IAPE-D and HERV-K, respectively). Data are the means ± standard deviations (s.d.) for at least three independent experiments. Bottom: retrotransposition frequency of an active autonomous IAP element marked with a *neo *indicator gene for retrotransposition [17] in the presence of the corresponding APOBEC3 proteins; the assay was performed by cotransfection of HeLa cells with the marked IAP and APOBEC expression vector as previously described [26]; values are the means ± standard deviations (s.d.) for at least three independent experiments and are given as percentages relative to the control (no apobec; 1.3 × 10^-3 ^G418^R ^clones/cell).

To assay whether the mouse IAPE element is restricted by APOBEC3 proteins, we used the previously described functional copy of IAPE-D (; mm9 July 2007 Assembly: chr12: 24,282,555–24,290,874) [18] that was cloned under the control of the CMV promoter, and in which a *neo *resistance gene was inserted in reverse orientation into the *env *gene (Figure 1A). The effect of APOBEC3 proteins on HERV-K was analyzed by using the "reconstituted" *Phoenix *element cloned under the control of the CMV promoter, in which the *env *gene is stopped and an anti-sense-oriented *neo *resistance gene is inserted into its 3'-LTR (Figure 1). Proviral clones of IAPE-D or HERV-K (4.5 μg), complemented with an expression vector for a functional IAPE or VSV-G Env (0.5 μg) respectively, and the murine (mA3) or human (hA3A-G) APOBEC3 proteins or a control plasmid (5 μg), were transfected in 293T cells. Supernatants were harvested 48 h post-transfection, filtered through 0.45-μm pore-size PVDF membranes, supplemented with Polybrene (4 μg/ml), and transferred onto HeLa target cells. To increase sensitivity, target cells were subjected to spinoculation at 1.200 × g for 2.5 h at 25°C. Infection events were detected after G418 selection of target cells and viral titers expressed as the number of G418^R ^clones per mL of supernatant. As illustrated in Figure 1, mA3 and hA3G protein expression leads to a dramatic decrease in both the IAPE-D and HERV-K viral titers (Figure 1B). In the case of the murine IAPE-D element, only a limited effect – if any – was observed with the human APOBEC3 proteins other than hA3G, with for instance no effect of hA3A which otherwise has a strong effect on the rate of retrotransposition of its intracellular counterpart, *i.e*. the IAP element (Figure 1). In the case of the human HERV-K, at variance with what is observed for the murine IAPE-D element, almost all the APOBEC3 proteins (with the exception of hA3C) have an effect, the highest activity being observed with hA3B and hA3F.

**Figure 2:**
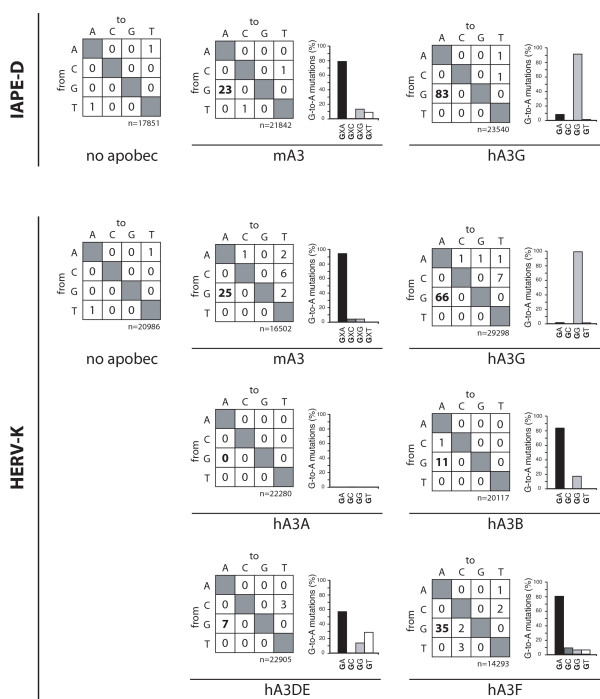
APOBEC3 proteins induce specific G-to-A hypermutations. Two-entry tables showing nucleotide substitution preferences in the presence of the indicated APOBEC3 proteins for the IAPE-D and HERV-K integrated proviruses. n, total number of bases sequenced. The adjacent graphs represent the relative frequencies of observed G-to-A mutations as a function of the G neighboring nucleotides (+2 position for the expected mA3 footprint, +1 position for the other APOBEC3s); for the two-entry tables, p-values calculated by a Poisson regression in a log-linear model for the occurrence of the G-to-A versus C-to-T mutations yielded p < 0.03 in all cases (except for hA3DE (p = 0.18) due to the low number of mutations); for the adjacent graphs, p-values calculated by a chi square test were p < 0.01 in all cases (except again for hA3DE, p = 0.7); similar levels of significance (or even higher) were obtained using the Kruskal Wallis test.

We further assessed whether the observed decrease in viral titers was associated with editing of the viral DNA by sequencing a 800 or 1600 bp fragment of the *de novo *integrated IAPE-D or HERV-K proviral DNA copies, respectively, in 20–25 individual G418^R ^clones. As illustrated in Figure 2 numerous G-to-A transitions were observed in the presence of mA3 or hA3G in both ERVs, as expected for an APOBEC3-mediated editing. For HERV-K, G-to-A editing was also observed with hA3B, hA3DE and hA3F, but not with hA3A, as expected from previous characterization of this enzyme ([3,21,24,29]; reviewed in [11]). Furthermore, mA3 and hA3G editing leads to G-to-A mutations in a GXA or GG context, respectively, which are the hallmarks previously described for each enzyme [26,31,32]. For hA3B and hA3F, G-to-A editing was observed in the GA context [2,33]. In addition, in spite of a low number of G-to-A mutations, hA3DE editing seems to preferentially take place in the GA/T context as expected [6]. It has to be stressed that the editing rate is probably underestimated because too heavily mutated *neo *genes present in these ERV DNAs can no longer confer G418 resistance after integration.

### Traces of APOBEC3 past activity on resident IAPE and HERV-K elements in the murine and human genome

**Figure 3:**
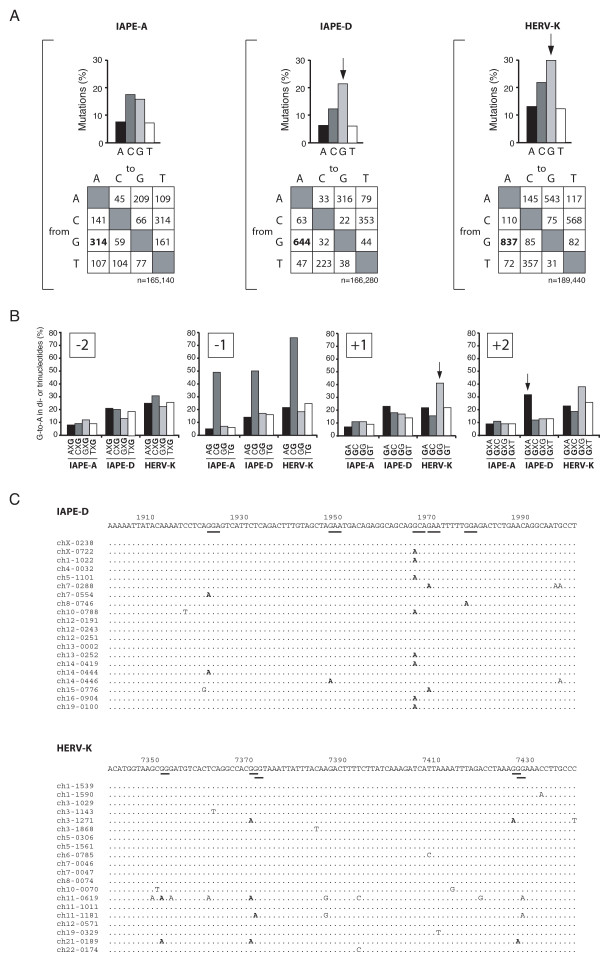
Distribution of the nucleotide substitutions in the IAPE-D and HERV-K genomic copies residing in the mouse and human genomes. Endogenous sequences were extracted from the mouse and the human genome databases, aligned and compared to the derived consensus. (A) Upper panels: percentage of substitutions for each nucleotide, for the endogenous IAPE-D and HERV-K elements (with the IAPE-A elements used as a control). Lower panels: two-entry tables showing nucleotide substitutions preferences, with the G-to-A values in bold (higher that the "non-specific" C-to-T value for IAPE-D and HERV-K, and identical in the case of the IAPE-A control). n, total number of nucleotides analyzed. (B) Influence of nucleotides at position -2, -1, +1 and +2 on G-to-A mutations (the mutated G is at position 0). Data represent the percentage of indicated target di- or trinucleotide sequences bearing G-to-A mutations. X represents any nucleotide. P-values calculated for the two-entry tables (in A) by a Poisson regression in a log-linear model for the occurrence of the G-to-A versus C-to-T mutations yielded p < 0.003 for IAPE-D and HERV-K; in B, p-values calculated by a chi square test were p < 0.001; similar levels of significance were obtained using the Kruskal Wallis test. (C) Example of G-to-A mutations present in twenty IAPE-D (upper panel) and HERV-K (lower panel) sequences. GXA trinucleotides and GG dinucleotides are underlined in the consensus sequence of IAPE-D and HERV-K, respectively.

Since the murine IAPE-D and the human HERV-K elements are found to be restricted by APOBEC3 proteins in the *ex vivo *assay above, we asked whether APOBEC3 proteins might have actually impaired the *in vivo *amplification of these elements in the past, by searching for evidence of APOBEC3-editing on the endogenous copies residing in the murine and human genome, respectively. Accordingly, an *in silico *analysis was performed to assess the levels of G-to-A mutations in two sets of full-length genomic IAPE elements, originating from two different subfamilies, namely IAPE-A and IAPE-D, and on full-length HERV-K elements. Both the murine IAPE-D subfamily and the human HERV-K elements have most probably been amplified by reinfection of the germline and therefore could have been subjected to APOBEC3 editing. Conversely, the IAPE-A subfamily has most probably been amplified via gene duplication, with several elements – essentially on the Y chromosome – disclosing identical flanking sequences [34,35], and therefore should not have undergone APOBEC3 editing: this family of elements – closely related to IAPE-D – can therefore be used as an internal control for the *in silico *genomic analyses. For all three families of elements, we selected by BLAST analysis a set of twenty copies displaying the closest sequence similarity to their cognate "master" copy: to the functional "Phoenix" element for HERV-K, to the functional copy used in the *ex vivo *assay for IAPE-D, and to the unique full-length copy with preserved open reading frames for IAPE-A. A consensus sequence was then derived for each family of elements, and each family member was analyzed for mutations to the consensus. As illustrated in Figure 3A, numerous mutations can be found for the three families of elements, consistent with the million years of genome evolution that have elapsed since the initial infection and/or amplification events. However, a specific increase in G-base mutations can be observed for both the IAPE-D and the HERV-K copies, not observed for the IAPE-A copies. These mutations are essentially G-to-A substitutions, with the effect being most probably "strand-specific", since the number of such mutations is almost twice that of the C-to-T substitutions. In addition, this bias is not observed for the IAPE-A elements, as expected for a duplicated element which has amplified by chromosomal DNA duplication, without a reverse transcription step prone to APOBEC3 mutagenesis. Interestingly, as illustrated in Figure 3B, the observed G-to-A changes are not randomly distributed but seem to be influenced by the neighbouring nucleotides: the GXA triplet is the most frequent "target" for the G-to-A substitutions in the IAPE-D elements (see arrow in Figure 3B), in agreement with previous reports – and data in Figure 2 – indicating that mA3 preferentially targets GXA trinucleotide motifs [26,31,33]. On the other hand, the G-to-A substitutions in the HERV-K copies are most frequently observed in the GG context (see arrow in Figure 3B), which corresponds to the footprint of hA3G editing [31] and data in Figure 2. There is no clear-cut evidence for G-to-A substitutions in the GA and GT context, excluding any significant contribution of hA3B, hA3DE or hA3F. Noteworthily, a "non-specific" bias can be observed for the endogenous IAPE-A, -D and HERV-K elements, which favors G-to-A mutations in CG dinucleotides (Figure 3B), most probably reflecting an APOBEC3-independent (since it is also observed for the duplicated IAPE-A elements) deamination of methylated-CpG islands. Finally, examples of sub-genomic regions of IAPE-D and HERV-K elements enriched in G-to-A substitutions, are shown in Figure 3C, where the di- or tri-nucleotide sequences specific for the hA3G and mA3 APOBEC proteins, respectively, are underlined. Altogether, these *in silico *data strongly suggest that the IAPE and HERV-K elements have been subjected to editing by specific APOBEC3 proteins during their retroviral cycle of amplification and insertion into their target host genome.

**Figure 4:**
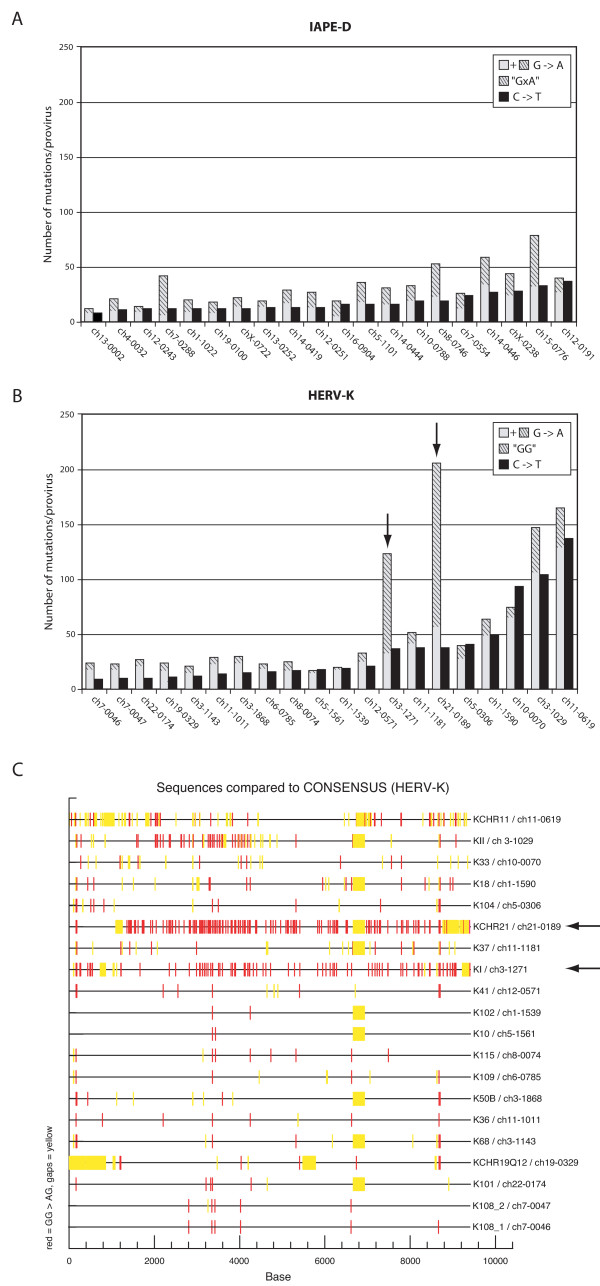
Variability of the number of G-to-A mutations within the endogenous IAPE-D and HERV-K proviruses depending on the element. The total numbers of G-to-A mutations (plain + hatched grey bars) for each IAPE-D (A) and HERV-K (B) proviruses are represented, together with that of the C-to-T (black bars) "none-strand-specific" mutations, given as an internal control (also used for ordering the elements) indicative of the genetic drift-associated age-dependent amount of mutations for each copy (same rank order as the sum of all non-G-to-A base substitutions). The number of G-to-A mutations specifically associated with the mA3 or hA3G APOBEC footprints is indicated with hatched grey. (C) Positioned G-to-A mutations (red bars) specifically associated with the hA3G APOBEC footprint ("GG") for the individual HERV-K elements in (B); yellow bars correspond to deletions in the proviruses (relative to the Phoenix consensus sequence). The data and the image shown in figure 4C were generated using the Hypermut 2.0 software available at the  website.

We further explored APOBEC3 editing by analyzing more specifically the G-to-A substitutions at the mA3 and hA3G target sites for each of the twenty IAPE-D and HERV-K proviruses, respectively. As shown in Figure 4A–B, for each proviral element, both the total number of G-to-A mutations (grey plus hatched grey) and the number of G-to-A mutations at the mA3- and hA3G-specific sites (hatched grey) were measured, together with the number of C-to-T "non-strand-specific" mutations as an internal control (dark bars; also used to order the copies in the Figure). Figure 4A–B then clearly shows that i) the total number of G-to-A mutations is for most proviruses higher than that of the "control" C-to-T mutations, ii) this increase is essentially due to "specific" mutations at the respective APOBEC3 sites, and iii) the extent of the observed mutations is highly variable depending on the proviral copy. Actually, for both the IAPE-D and HERV-K proviruses, more G-to-A mutations than C-to-T mutations can be observed, consistent with a strand specificity that can only have occurred prior to integration; in addition, this excess of G-to-A mutations is in general observed at GXA triplet positions for the murine, mA3-sensitive IAPE-D (> 40% of the G-to-A mutations for the majority of the proviruses, namely thirteen out of twenty), and at GG doublet positions for the human, hA3G-sensitive HERV-K elements. For the latter, it should be noted that the extent of specific G-to-A mutations is rather limited (seventeen out of the twenty HERV-K proviruses display < 30% of their G-to-A mutations at GG positions), except for two proviruses (ch3-1271 and ch21-0189) which are specifically hypermutated (Figure 4B–C), with > 70% of their G-to-A mutations in the GG context, without any evidence for a clear-cut gradient along the proviral sequence (Figure 4C). These results indicate that HERV-K can indeed be severely edited by hA3G, and that APOBEC3G protein expression at different times of HERV-K amplification in the human genome must have been quite variable.

## Conclusion

The restriction effects of APOBEC proteins on endogenous retroelements have essentially concerned retrotransposons with a strictly intracellular life cycle, namely the LINE/SINE non-LTR retrotransposons, and LTR-retrotransposons including the yeast Ty1 element [36,37], and the IAP and MusD murine elements [3,24,26,33]. In these cases severe restriction has been observed, both in *ex vivo *assays and by *in silico *analysis of the traces that APOBEC proteins have left through DNA edition in the course of reverse transcription of the retroelements [26]. Here we show that similar effects take place at the level of endogenous retroviruses with an extracellular life cycle, with an unambiguous restriction of the murine IAPE by a murine APOBEC3 protein, and of the human HERV-K element by a human APOBEC3 protein. Taking into account that an infectious IAPE retrovirus with an extracellular life cycle has been the progenitor of the IAP element, the restriction observed for IAP by mA3 appears simply to be the consequence of the restriction that initially controlled the progenitor infectious IAPE invading the rodent ancestor, with the effect being maintained in the evolution of the endogenized IAP retroelements. Although it concerns a heterologous – and therefore not necessarily very relevant-situation, it is noteworthy that the human hA3A protein can control the murine intracellular IAP retroelement, a property not observed for the IAPE infectious progenitor. This is most probably relevant to the localization of the hA3A protein – in the nucleus – and to its rather atypical mode of action – not involving editing of the reverse transcribed DNA – which identifies this restriction factor as more specifically devoted to intracellular retrotransposons, consistent with the absence of reported effects of this factor on – most – infectious retroviruses (reviewed in [2]). Finally, *in silico *analysis of the genomic copies of the elements demonstrates that APOBEC3 editing has taken place in evolution for these amplified elements, with clear-cut evidence for a severe heterogeneity in the extent of the editing process.

## Methods

### Plasmids

The human (HERV-K) and murine (IAPE-D) *neo*-marked ERV copies (pBS CMV-Kcons Stop Env neoAS and pCMV RU5 IAPE neoAS, respectively), the VSV-G and IAPE-D *env *expression vectors, and the *neo*-marked autonomous murine IAP retrotransposon (pGL3-IAP92L23 neo^TNF^) have been previously described [17-19]. The APOBEC3 expression plasmids were obtained from M. Malim (hA3A), the NIH AIDS Research and Reference Reagent program (hA3B, hA3C and hA3F), Open Biosystems (hA3DE), A. Hance (hA3G), and N. Landau (mA3). A plasmid expressing a defective hA3G gene (with a premature stop codon) was used as a negative control. All the APOBEC3 ORF-containing fragments were re-cloned into the pcDNA6 expression plasmid (Invitrogen).

### Retrotransposition and infection assays

Retrotransposition assays with the neo-marked IAP were as described previously [38]. For the infection assays, 293T cells seeded in 60-mm-diameter plates were transfected using the Lipofectamine Plus kit (Invitrogen) with 4.5 μg of the *neo*-marked *env*-defective murine or human ERV, 0.5 μg of the IAPE or VSV-G *env *expression vector, and 5 μg of the APOBEC3 expression vector to be tested. Supernatants were harvested 48 h post-transfection, filtered through 0.45-μm pore-size PVDF membranes, supplemented with Polybrene (4 μg/ml), and used to infect HeLa target cells by spinoculation (1.200 g for 2.5 h at 25°C). Infection events were detected upon G418 selection of target cells and viral titers quantified as the number of G418^R ^clones per mL of supernatant [18,19].

### Analysis of integrated proviral DNAs

Cellular DNA from 20–25 individual G418^R ^clones was used to PCR-amplify a 996 bp fragment encompassing the *env *to *neo *gene region (nt 6783–7779) of the IAPE-D element and a 2049 bp fragment spanning the *neo *to *gag *gene region (nt 1093–3142) of the HERV-K element (initial 3 min denaturation step at 94°C; 40 cycles: 94°C, 50 sec; 60°C, 50 sec; 68°C, 150 sec). PCR reactions were performed with sets of appropriate primers in 50 μl containing 0.5 μg of cellular DNA, 1× Buffer II and 1.5 U AccuPrime *Taq *DNA polymerase (Invitrogen). The PCR products were electrophoresed on agarose gels, purified with the Nucleospin Extract II kit (Macherey-Nagel) and a ~800 bp or a ~1600 bp fragment was sequenced (Applied Biosystem sequencing kit) for IAPE-D and HERV-K, respectively.

### Human and Mouse genome analyses

IAPE-D, IAPE-A and HERV-K endogenous retroviruses were extracted from the mouse and human genome sequence databases (Mouse GoldenPath mm8, February 2006 assembly and Human GoldenPath hg18, March 2006 assembly; ) by using as a querying probe the sequence of the previously described functional IAPE-D1 copy [18], the sequence of the IAPE-A copy with intact *gag*-*pol *open reading frames (chr14-0436, [18]), and the sequence of the HERV-K element (Phoenix-derived; [19]) used in the cell-based infection assay. Twenty sequences displaying the highest homology to their cognate probe were selected for the IAPE-D and HERV-K elements. Twenty sequences with the highest homology to the IAPE-A sequence and localized on the Y chromosome were also selected to be used as a control (see Results). Alignments were performed using the ClustalW and Editsequence softwares and consensus sequences generated. Quantitative analysis of the nucleotide substitutions within the IAPE-A, IAPE-D and HERV-K elements was performed using Excel and Hypermut 2.0 (available at the  website) softwares, on the full-length retroviruses. The localization of the analyzed sequences within the mouse and human genomes are given in additional file 1.

### Statistical analyses

Significance levels for the data in Figures 2 and 3 were calculated using the Kruskal Wallis test (GrapPrism software package). More refined analyses for the occurrence of the G-to-A versus C-to-T mutations were performed using a Poisson regression in a log-linear model. The genmod procedure of the SAS software was used (version 9.1, SAS Institute Inc, Cary, NC). The observed distributions of the G-to-A mutations among the GA, GC, GG and GT contexts for HERV-K or the GXA, GXC, GXG and GXT contexts for IAPE were compared to the distribution of these di- or trinucleotides by the chi square test.

## Competing interests

The authors declare that they have no competing interests.

## Authors' contributions

CE, SP and DR carried out the experimental work and drafted the manuscript. OH performed the *in silico *analyses and drafted the manuscript. TH conceived the study and drafted the manuscript. All authors read and approved the final manuscript.

## Supplementary Material

Additional file 1 table 1. localization of the analyzed sequences within the mouse and human genomes. Click here for file
