# Supplementary material for: Restriction by APOBEC3 proteins of endogenous retroviruses with an extracellular life cycle: ex vivo effects and in vivo "traces" on the murine IAPE and human HERV-K elements
Source: Retrovirology. 2008 Aug 14;5:75. doi: 10.1186/1742-4690-5-75 (PMC2531183; doi:10.1186/1742-4690-5-75)
Supplement: Additional file 1 — table 1. localization of the analyzed sequences within the mouse and human genomes. [file 1742-4690-5-75-S1.pdf]

IAPE-D Build 37 (mm9 assembly) july 2007

chX-0238	chrX : 23730557-23739257
chX-0722	chrX : 72108847-72125669
ch1-1022	chr1 : 102120400-102128717
ch4-0032	chr4 : 3119359-3127677
ch5-1101	chr5 : 110073813-110082133
ch7-0288	chr7 : 28718765-28727084
ch7-0554	chr7 : 55379494-55387811
ch8-0746	chr8 : 74548682-74556981
ch10-0788	chr10 : 78777007-78785319
ch12-0191	chr12 : 19055098-19063398
ch12-0243	chr12 : 24282555-24290874
ch12-0251	chr12 : 25042010-25049933
ch13-0002	chr13_random : 152862-161181
ch13-0252	chr13 : 25099269-25107588
ch14-0419	chr14 : 41866691-41874954
ch14-0444	chr14 : 44382039-44390353
ch14-0446	chr14 : 44502824-44511371
ch15-0776	chr15 : 77514301-77522565
ch16-0904	chr16 : 90341789-90350117
ch19-0100	chr19 : 9903338-9911654

IAPE-A Build 36 (mm8 assembly) febr 2006
All 20 sequences were from chrY_random at positions :

14184286-14192566
1571264-1579519
2421063-2429355
9186724-9194979
561072-569325
12258547-12266836
11181590-11189847
10609812-10618074
12633892-12642147
9694801-9703014
13203818-13212072
3788682-3796936
2665670-2673871
12407502-12415713
1362196-1370415
10288055-10296239
13016410-13024603
2118927-2127152
12793664-12801852
8722652-8730938

HERV-K Build 36.1 (hg18 assembly) march 2006

ch1-1539	chr1 : 153863081-153872260
ch1-1590	chr1 : 158927199-158936430
ch3-1029	chr3 : 102893427-102902549
ch3-1143	chr3 : 114225814-114234972
ch3-1271	chr3 : 127091992-127101129
ch3-1868	chr3 : 186763030-186772209
ch5-0306	chr5 : 30522517-30531962
ch5-1561	chr5 : 156017295-156026474
ch6-0785	chr6 : 78483381-78492802
ch7-0046	chr7 : 4588583-4598054
ch7-0047	chr7 : 4597087-4606557
ch8-0074	chr8 : 7342807-7352269
ch10-0070	chr10 : 6906147-6915609
ch11.0619	chr11 : 61892539-61907139
ch11-1011	chr11 : 101071004-101080469
ch11-1181	chr11 : 118096934-118106093
ch12-0571	chr12 : 57007509-57016965
ch19-0329	chr19 : 32820338-32829224
ch21-0189	chr21 : 18855302-18863833
ch22-0174	chr22 : 17306187-17315361
